# Predictive combinatorial design of mRNA translation initiation regions for systematic optimization of gene expression levels

**DOI:** 10.1038/srep04515

**Published:** 2014-03-31

**Authors:** Sang Woo Seo, Jae-Seong Yang, Han-Saem Cho, Jina Yang, Seong Cheol Kim, Jong Moon Park, Sanguk Kim, Gyoo Yeol Jung

**Affiliations:** 1 Department of Chemical Engineering, Pohang University of Science and Technology, San 31, Hyoja-dong, Nam-gu, Pohang, Gyeongbuk, Korea, 790-784; 2 School of Interdisciplinary Bioscience and Bioengineering, Pohang University of Science and Technology, San 31, Hyoja-dong, Nam-gu, Pohang, Gyeongbuk, Korea, 790-784; 3 Division of Molecular and Life Science, Pohang University of Science and Technology, San 31, Hyoja-dong, Nam-gu, Pohang, Gyeongbuk, Korea, 790-784; 4 Division of IT Convergence Engineering, Pohang University of Science and Technology, San 31, Hyoja-dong, Nam-gu, Pohang, Gyeongbuk, Korea, 790-784; 5 These authors contributed equally to this work.

## Abstract

Balancing the amounts of enzymes is one of the important factors to achieve optimum performance of a designed metabolic pathway. However, the random mutagenesis approach is impractical since it requires searching an unnecessarily large number of variants and often results in searching a narrow range of expression levels which are out of optimal level. Here, we developed a predictive combinatorial design method, called UTR Library Designer, which systematically searches a large combinatorial space of expression levels. It accomplishes this by designing synthetic translation initiation region of mRNAs in a predictive way based on a thermodynamic model and genetic algorithm. Using this approach, we successfully enhanced lysine and hydrogen production in *Escherichia coli*. Our method significantly reduced the number of variants to be explored for covering large combinatorial space and efficiently enhanced pathway efficiency, thereby facilitating future efforts in metabolic engineering and synthetic biology.

Balancing expression levels between genes encoding pathway enzymes is a prerequisite for achieving optimized performance of the designed metabolic pathway^1^,^2^. Imbalances among pathways often cause a toxic accumulation of metabolic intermediates that may pose an undue metabolic burden and result in failed production of target products. However, it still remains a challenge to develop reliable and precise methods for exploring broad expression levels of pathway enzymes in a predictive manner to increase the pathway efficiency.

Expression levels of genes along a given pathway have typically been altered using overexpression or knockout strategies; however, optimal expression levels usually lie somewhere between these extremes^1^,^3^. Although promoter engineering has been widely used to modulate gene expression at the transcriptional level^4^, it is also necessary to control mRNA structural elements around the translation initiation region (TIR) including the 5′-untranslated region (5′-UTR) and 5′-proximal coding sequence of mRNAs. These mRNA structural elements have a great impact on gene expression levels, especially in cases where inherent regulatory features or design constrains make it impractical or impossible to further modify promoter regions^5^,^6^. However, performing random mutagenesis of the 5′-UTR to investigate optimal expression levels without knowledge of mutations that specifically affect expression levels is impractical because it normally results in sampling of small range of solution space and requires exploration of exceedingly large libraries. These impractical combinations of mutations might limit our ability to search large solution space and consequently yield few beneficial phenotypes by exhausting extremely high costs due to the current technical limitations in library generation as well as screening/selection throughputs^7^. Thus, a novel method that can explore expression levels across a broad range while minimizing the number of mutations is required to generate a practical library that covers a large, but feasible, space for performing systematic combinatorial optimization of pathway efficiency.

In this study, we developed a method, called UTR Library Designer, for the combinatorial design of TIR based on a thermodynamic model and genetic algorithm to facilitate systematic optimization of gene expression levels. This method generates TIR sequences (one-to-many) that varies gene expression levels between a minimum and maximum level with a selected number of intermediate points. Furthermore, we applied this method to enhance the lysine and hydrogen productivities by controlling expression levels of *ppc* and *gapA*, respectively. Our approach enabled us to efficiently enhance the efficiencies of metabolic pathways compared to random mutagenesis.

## Results

### Model-driven combinatorial design of mRNA TIR

**Figure 1:**
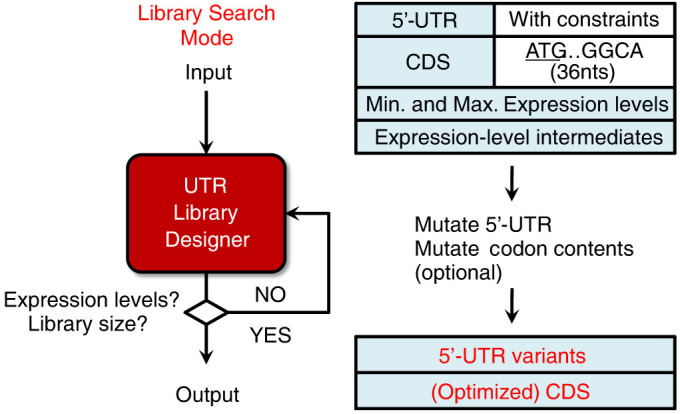
UTR Library Designer. UTR Library Designer designs the 5′-UTR variants to yield a specific target range of expression levels with a selected number of intermediate points by mutating the 5′-UTR sequences or, when variations in 5′-UTR do not satisfy the desired expression levels, altering the codon content of the coding sequences.

We developed UTR Library Designer to search the combinatorial space of gene expression levels by designing mRNA TIR sequence that covers a desired range of gene expression levels (Fig. 1). The design solution was optimized by applying a thermodynamic energy model to a genetic algorithm. Briefly, our thermodynamic energy model (ΔG_UTR_) calculates the difference in Gibbs free energy before and after the 30S complex assembles onto an mRNA transcript by considering ribosome binding affinity and accessibility to mRNA^5^. Using a linear relationship between (ΔG_UTR_) and log expression level, we can design sequences that meet a specific expression level. We used this relationship for the forward engineering mode of UTR Designer with the genetic algorithm. Briefly, an initial sequence population was randomly generated and the fitness of each sequence was evaluated by the difference between desired and predicted expression levels. Only top *n* closest sequences in expression with desired level can be remained in the population and others are eliminated. Then, each remained sequence is changed with little mutations on its sequence. These selection and mutation steps were conducted until a sequence was found that has desired level of expression.

**Figure 2:**
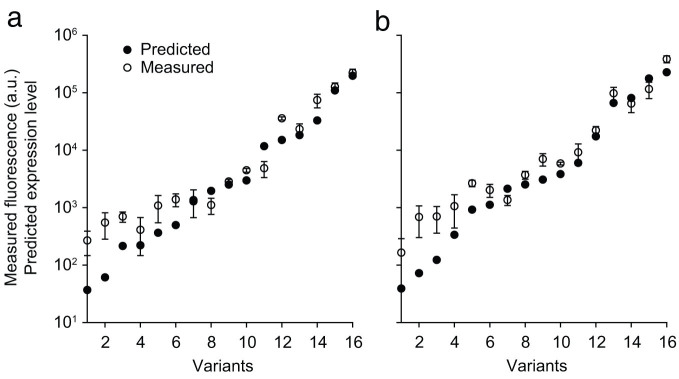
*In silico* and *in vivo* expression levels of libraries with 16 expression-level intermediates using *sgfp* fluorescent reporter. Two different 16-bp library sequences were used for validation assay. The *y*-axis indicates log fluorescence measured and predicted expression level. Variants of 5′-UTR sequences are as follows: (a) CCTRTTGTCTAAAGKAGSATCGCCM and (b) GCTGMCAGAGAAAGSAGCRTCMTTG. Experiments were performed in triplicate and error bars indicate standard deviation.

UTR Library Designer also utilized this energy model to generate 5′-UTR variants to achieve a desired range of gene expression levels. The difference between UTR Designer and UTR Library designer is that UTR Library designer finds minimum- and maximum-expression sequences at the same time and has post-analysis step to find desired number of intermediates. The algorithm has been optimized to find sequences that can change its expression level from desired maximum to minimum expression levels by the limited number of mutations (Supplementary Fig. 1). Specifically, our method takes a 5′-UTR template and coding sequence, desired minimum/maximum expression levels, and constraints (e.g., expression-level intermediates, nucleotide constraints) for design. Our method finds sequences that could generate desired maximum expression level and minimum expression level with genetic algorithm described above with *n* = 1, separately. Then, it mutates nucleotide sequences in the 5′-UTR to create a pool of mRNA sequences to analyze the effect of mutation in a certain position of the sequences. For example, it ranks mutation positions that change expression levels from major to minor amount. If mutated sequences for maximum and minimum expression achieve the desired levels, the two sequences are combined into a sequence capable of covering a diverse range of expression. As such, the algorithm selectively adds mutations to match the desired number of mutations. After several different trials, the algorithm gives the best solution. Since UTR Library Designer employs a genetic algorithm that mimics an evolutionary process to search optimal sequences in nature^8^, it reaches a desired range of gene expression levels much faster than random trials. For example, UTR Library Designer easily achieved 5′-UTR variants yielding 5,000-fold changes in expression level with 16 expression-level intermediates, which is an extremely rare event (4 in 50,000 trials) using random sequences (Supplementary Fig. S2 and Supplementary Tables S1 and S2). Even when applied to comparisons of greater than 2,000-fold changes, UTR Library Designer is at least 200 times faster than random trials (Supplementary Table S3). We tested two different libraries with 16 expression-level intermediates obtained above using green fluorescent protein (*sgfp*) *in vivo*. We could observe that the fluorescence level *in vivo* was matched well with the predicted expression level *in silico* (Fig. 2).

### Verification of UTR Library Designer for library generation

**Figure 3:**
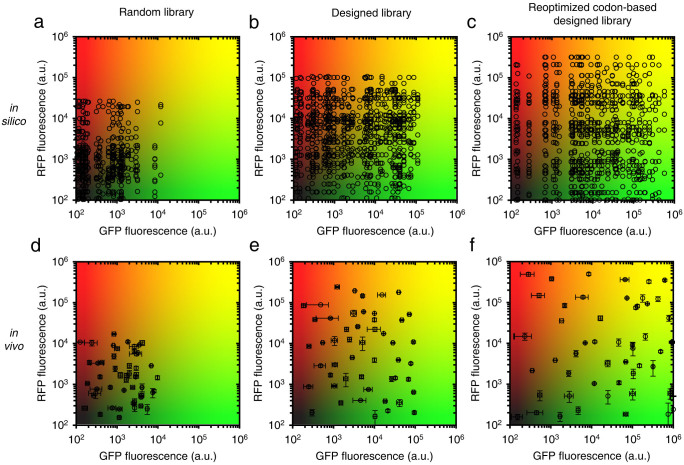
Verification of UTR Library Designer for library generation. The range of expression levels of 5′-UTR libraries containing 128 sequences for each reporter gene generated by different design methods ((a) and (d): random library; (b) and (e): designed library; (c) and (f): reoptimized codon-based designed library) was examined both *in silico* (a–c) and *in vivo* (d–f). For the *in silico* analysis, 1,000 events were randomly sampled out of a possible 16,384 libraries for each case. For the *in vivo* analysis, 50 clones randomly selected from agar plates were grown, and fluorescence for each case was measured. Experiments were performed in triplicate and error bars indicate standard deviation.

The ability of UTR Library Designer was also validated by designing large number of variants using fluorescent proteins. Using non-optimized coding sequences of two different fluorescent reporters (green and red fluorescent proteins encoded by *gfp* and *rfp*), we designed 5′-UTR libraries containing 128 sequences for each reporter gene to have broad range of expression levels (designed library) and compared them with random library which randomize consecutive five nucleotides around the Shine-Dalgarno (SD) sequence (Supplementary Fig. S3). The maximum expression level covered by these libraries (designed library) was predicted to be 3- to 10-fold higher than that of libraries generated by consecutive random mutations (random library) (Supplementary Fig. S3b and c). When we randomly sampled 1,000 variants among all possible combinations of *gfp* and *rfp* libraries *in silico*, the range of expression levels covered by designed library was predicted to be much larger than that of random library (Fig. 3a and b). Furthermore, combinatorial design after additional codon optimization of the N-terminal region of each reporter gene further increased the predicted maximum expression level of the libraries (reoptimized codon-based designed library) by 3- to 5-fold compared to that of designed library (Supplementary Fig. S3a). Consequently, the difference was 10- to 50-fold higher than that of random library. Thus, the predicted expression-level space covered by reoptimized codon-based designed libary was much larger even than that of designed library (Fig. 3c).

**Figure 4:**
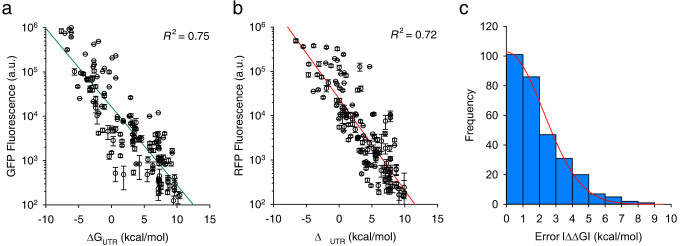
Correlation between ΔG_UTR_ and expression level. The ΔG_UTR_ for each variant library showed a linear correlation with expression level for (a) *sgfp* and (b) *mCherry* genes, as expected. (c) Distribution of the error (|ΔΔG|) in histogram from (a) and (b). The average of the distribution is 1.98 kcal/mol and fits well to a one-sided Gaussian distribution (red line) with s.d. σ = 1.60 kcal/mol.

To verify the utility of UTR Library Designer *in vivo*, we analyzed three combinations of *gfp* and *rfp* libraries (random, designed, and reoptimized codon-based designed libraries) by co-transformation of two plasmids simultaneously. As shown in Materials and Methods, we first conducted low-throughput analysis by manually isolating 50 clones of each case that have expression levels as broad as possible based on the fluorescence colors. They showed that the patterns of expression-level coverage were similar to those from *in silico* analysis (Fig. 3d–f). Each fluorescent protein variant showed a linear relationship between the ΔG_UTR_ predicted by UTR Designer and log fluorescence intensities, and the average error was 1.98 kcal/mol with standard deviation of 1.60 kcal/mol (Fig. 4). To observe the entire population, we analyzed non-isolated clones by using two-color FACS. The difference in expression levels between libraries was similar to that expected from *in silico* analysis as well as low-throughput analysis. The range of expression levels of reoptimized codon-based designed library (Supplementary Fig. S4c) was larger than that of random library (Supplementary Fig. S4a) and even than that of designed library (Supplementary Fig. S4b). Although we also found non-uniform density of sampling points between libraries from FACS analysis (Supplementary Fig. S4), we believe that it is originated from experimental artifact during library construction because the higher density region is concentrated at the lower expression level. It can be due to either transformation of plasmid library with non-functional fluorescent protein (error caused by library preparation during PCR) or non-uniform synthesis of degenerate oligonucleotides. Nevertheless, these results indicate that our method facilitates the investigation of specific expression-level combinatorial-space in a predictive manner.

### Enhanced pathway efficiency driven by UTR Library Designer

Next, we applied UTR Library Designer to control the expression level of a gene encoding one of the pathway enzymes that plays a key role in determining pathway efficiency. When the high-throughput colorimetric, fluorescent, and growth-coupled screening methods are available, the number of expression-level intermediates of library can be large enough depending on the number of genes along the pathway to cover a larger feasible space. However, when the throughput of screening is limited, it should be low enough to verify the performance of the constructed variants.

**Figure 5:**
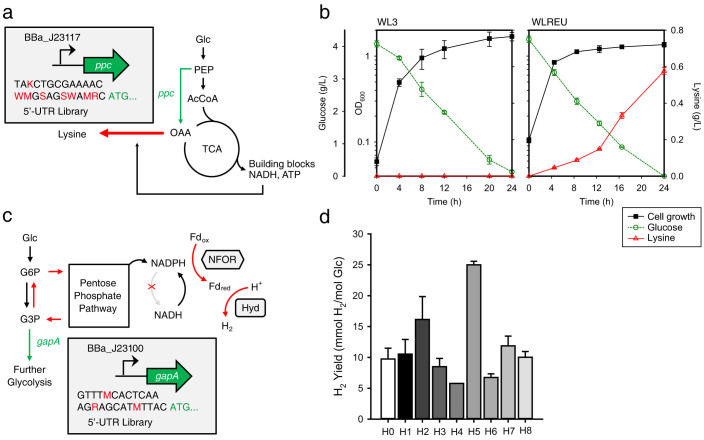
Pathway optimization driven by UTR Library Designer. (a) Simplified metabolic pathway leading to lysine production in *E. coli*. The expression level of *ppc* was controlled by using 5′-UTR variants. Base symbol usage follows the International Union of Pure and Applied Chemistry (IUPAC) system. Glc, glucose; PEP, phosphoenolpyruvate; AcCoA, acetyl-CoA; OAA, oxaloacetate. (b) Physiological comparison between the parental strain (WL3, left panel) and the enriched strains (WLREU, right panel). The left *y*-offset and right *y*-axis represent glucose (green open circles with dashed line) and lysine (red open triangles with solid line) concentration (g/L), respectively. The left *y*-axis represents optical density (black closed rectangles with solid line) at 600 nm in log scale. The *x*-axis represents the culture time (h). Experiments were performed in triplicate and error bars indicate standard deviation. (c) Simplified metabolic pathway leading hydrogen production in *E. coli*. The expression level of *gapA* was controlled by using 5′-UTR variants. Glc, glucose; G6P, glucose-6-phosphate; G3P, glyceraldehyde-3-phosphate; Hyd, NADPH-dependent-[FeFe]-hydrogenase; Fd, ferredoxin; NFOR, NAD(P)H:ferredoxin oxidoreductase. (d) Hydrogen production yield of the designed variants. The *y*-axis represents H_2_ yield per mole of glucose and the *x*-axis represents the variants. Experiments were performed in triplicate and error bars indicate standard deviation.

First, we chose the lysine metabolic pathway, seeking to balance the flux distributions of anaplerotic and glycolytic pathways around the phosphoenolpyruvate (PEP) node using a high-throughput screening method based on the Lysine Riboselector comprised of a lysine-responsive riboswitch and a selection marker gene to enable cells showing high lysine production to survive under selection pressure^9^. To achieve this, we attempted to determine the expression level of *ppc* for increased lysine production by combinatorially designing 5′-UTR libraries (256 variants) of *ppc* encoding PEP carboxylase, a key anaplerotic enzyme, yielding more than a 10^5^-fold range of expression levels (Fig. 5a; Supplementary Fig. S5). As noted, the library was large enough to cover a broader, but feasible, space since a high-throughput screening method was available. After three rounds of screening process as in our previous study^9^, twenty colonies were randomly selected, and the plasmids were extracted and sequenced. All isolated clones had the same 5′-UTR sequence at the region upstream of *ppc* without any selective mutations in the promoter region, and the expression level of the particular sequence was predicted to be in approximately the middle of the range of the entire library (Supplementary Fig. S5). As expected, this enriched strain (WLREU) showed a dramatic increase in lysine production compared to the parental strain (WL3), which showed very little lysine accumulation in the culture broth (Fig. 5b). Interestingly, the ability of the strain to produce lysine was similar to those of the strains previously enriched from a promoter library^9^. There are two possible reasons why we could isolate only one variant out of library. When we used promoter libraries in the previous study, we could isolate three different clones that showed similar lysine production. However, in that case, we used 10^7^ size of library that might cover the range of expression level in a finer way than these 256 variants from 5′-UTR modifications, and thus there could be only one candidate clone that can satisfy the cutoff level of lysine production. Also, since our model is not 100% accurate (*R*^2^ = 0.7–0.8), it is plausible that there might be additional factors determining gene expression level besides the binding energy calculation given that only one particular sequence was enriched even if there are other potential sequences predicted to have similar binding energies. Collectively, these results indicate that a particular strain with a *ppc* expression level for increased lysine production can also be successfully enriched through predictive library design of the 5′-UTR.

Finally, we applied our method to the biological production of hydrogen, which has been intensively studied even in the absence of an appropriate screening system. Of the various biological hydrogen production methods, we chose to implement a dark fermentation system by coexpressing NADPH-dependent-[FeFe]-hydrogenase (Hyd), ferredoxin (Fd), and NAD(P)H:ferredoxin oxidoreductase (NFOR) in which protons are reduced to hydrogen through electron transfer using NADPH generated by the pentose phosphate pathway (Fig. 5c)^10^,^11^. In order to modify the flux around glyceraldehyde-3-phosphate node, we used the previously established glycolysis shut-down system^12^ and attempted to control the expression level of *gapA* encoding glyceraldehyde-3-phosphate dehydrogenase (GAPDH). The designed 5′-UTR library (8 variants) was predicted to yield more than a 100-fold range of expression levels (Supplementary Figure S6a). In addition, the specific enzymatic activity produced by each variant was linearly correlated with the predicted expression level even if four data points at the extreme low end are removed (*R*^2^ = 0.64 without data at the low end and *R*^2^ = 0.91 with all data points; Supplementary Fig. S6b). When each strain was cultured, the hydrogen formation was not linearly correlated with the activity of GAPDH in this particularly designed system and rather had local maxima in H2 and H7 strains and the highest maximum in H5 strain, indicating that hydrogen production shows dramatic non-linear behavior with changes in *gapA* expression levels as other systems did^1^ (Fig. 5d). The one variant (H5) showed a 2.5-fold increase in the yield of hydrogen production. The total amount of hydrogen evolved in the H5 strain was also about 4-fold higher than that of the control strain (H0) (Supplementary Fig. S6c). These results indicate that the increase in yield was not due to a retarded growth rate or reduced glucose consumption rate of the H5 strain. Interestingly, although the difference of the predicted expression level between H5 and H6 strains was around 30% and that of the measured activity was around 20%, they showed completely different capacity of hydrogen production meaning that this change was substantial for controlling the metabolic flux in the cell.

## Discussion

Identifying biologically relevant maximum and minimum levels of enzyme expression and exploring the optimal level between them is a key to successful optimization of enzyme expression for performance of designed tasks^1^,^3^. However, applying a random approach to solve this long-standing issue is often impractical because of two reasons. First, the designable number of variants for optimizing expression level is limited when relying on a random approach because it is virtually impossible to cover such a large solution space. Second, the size of the potential solution space dramatically exceeds the physiologically obtainable search space once the number of mutations are increased^7^. However, in this study, we showed that our method, UTR Library Designer, could vary expression levels of a target gene across a broad range, while minimizing the number of mutations, through generation of 5′-UTR variants and further optimization of 5′-proximal coding sequences (TIR).

Because of its model-driven library design, our method could be used to examine a broad range of expression levels of target genes (*ppc* and *gapA*) to enhance pathway efficiencies. In case of *in silico* analysis, UTR Library Designer generated sequence libraries that achieved 10^5^- and 10^2^-fold expression changes for *ppc* and *gapA*, respectively. In contrast, with the same number of expression-level intermediates as used for UTR Library Designer (256 for *ppc* and 8 for *gapA*), a random approach that generated 10,000 different sets of library pools was largely unable to achieve such expression changes from *in silico* analysis (*P*-value < 10^−4^, Supplementary Fig. S7 and S8). Moreover, the probability of the random library including a variant with an expression level similar (95%–105%) to that of the optimal value obtained was approximately 10% (11.31% for *ppc* and 5.25% for *gapA* out of 10,000 library pools), indicating that our method efficiently facilitated achieving the specific value.

Since pathway optimization requires fine-tuning of expression levels over a subtle range^1^, our method could be used for a grid search with a broad range of expression levels to find a sub-optimal expression level in the first round of the screening process, while providing an opportunity for additional search by further narrowing the range of expression levels to be explored in subsequent screening rounds. When considering a complicated pathway composed of several different enzymes, it is necessary to optimize multiple target genes through simultaneous searches of each gene's expression level. In contrast to random approaches, our method would be surprisingly efficient to apply in such cases because it does not rely on searching unpredictable random sequences, which could result in a combinatorial explosion. In addition, designing 25-bp 5′-UTR variants without additional genetic components can reduce failures that arise from using repetitive sequences during the generation of libraries^13^,^14^. Since robust methods for assembling DNA constructs and editing genomic DNA with high efficiency are readily available^15^,^16^, our method could plausibly be used to optimize the expression of multiple enzymes simultaneously for various purposes by covering a practical range of library space.

## Methods

### Software implementation

A software implementation of the combinatorial design method developed in this study (UTR Library Designer) is available on our web server (http://sbi.postech.ac.kr/utr_library). Users can apply this software to generate 5′-UTR variants and optimized coding sequences (optional within the same codon preference) to meet a specific target range of expression levels with a selected number of intermediate points. Users have to input template 25-bp 5′-UTR sequences and constraints for designing 5′-UTR variants (or default sequences) as well as at least 35-bp of coding sequence after start codon. We suggest the use of a proportional range of 1 to 1,000,000, although a wider range is also potentially feasible for this system. The software provides outputs depending on the sequences and the number of expression-level intermediates that users have input. We recommend to reoptimize codon contents with same codon preference on our web server when the 5′-UTR variants fail to satisfy the desired range of expression levels.

### Reagents, bacterial strains, plasmids, and primers

*Phusion* polymerase and restriction endonucleases were purchased from New England Biolabs. pACYC-Duet, pCDF-Duet, and pET-Duet vectors were purchased from Novagen. The *E. coli* bacterial strains and plasmids used in this study are listed in Supplementary Table S4. The oligonucleotides used for the construction of plasmids and libraries were synthesized by Bioneer (Daejeon, Korea) and are listed in Supplementary Table S5. All other reagents were obtained from Sigma unless otherwise indicated.

### Construction of the 5′-UTR library and strains

pCDF-mCherry and pCDF-mCherryOpt plasmids were generated by amplifying pCDF-Duet using the 5′-phosphorylated pCDF-M-F-P and pCDF-pET-M-R-P primers followed by blunt-end ligation. The internal *Xba*I site was removed by site-directed mutagenesis using the pCDF-del-XbaI-F and pCDF-del-XbaI-R primers, as described in a previous study^17^. The mCherry and mCherryOpt genes were amplified using XbaI-mCherry-F/SphI-mCherry-R and XbaI-mCherryOpt-F/SphI-mCherry-R primer pairs, respectively, and were inserted into the *Xba*I and *Sph*I sites of the modified pCDF vector. To test algorithm's ability, we generated 5′-UTR libraries with 16 expression-level intermediates by using pACYC-sgfpOpt as a template in polymerase chain reactions (PCR) employing 5′-phosphorylated primers. Each PCR mixture consisted of 50 ng of template, 10 pmol of each primer, 0.5 U *Phusion* DNA polymerase, 250 mM each dNTP, 10 μl of the 5× buffer provided by the manufacturer, and H_2_O to a final volume of 50 μl. Reactions were carried out on an Applied Biosystems Thermal Block (Applied Biosystems, Foster City, CA, USA) under the following conditions: 30 s at 98°C followed by 20 cycles of 10 s at 98°C, 15 s at an annealing temperature determined based on the T_m_ of the primers and 3 min at 72°C, followed by a final extension at 72°C for 10 min. The resulting PCR products were purified using a QIAquick PCR Purification Kit (Qiagen GmbH, Germany), and the template DNA was eliminated by treating with *DpnI* at 37°C for 1 h. The PCR products were blunt-end ligated using T4 DNA ligase (TaKaRa, Kyoto, Japan) at 16°C overnight, and then used to transform the *E. coli* ElectroMAX DH5α-E strain (Invitrogen, Carlsbad, CA, USA). Purified plasmids were sequenced by Solgent (Daejeon, Korea) using an ABI 3730XL capillary DNA sequencer. In case of 5′-UTR variants for random, designed, and reoptimized codon-based designed searches of fluorescent proteins, we used each constructed plasmid as a template—pACYC-sgfp and pCDF-mCherry for random/designed searches; pACYC-sgfpOpt and pCDF-mCherryOpt for reoptimized codon-based designed searches. Other steps were same as described above except for co-transformation of two different plasmid libraries (sgfp and mCherry) into a same competent cell simultaneously.

The 5′-UTR library for *ppc* was constructed by PCR-based blunt-end ligation with 5′-phosphorylated ppc-UTR-lib-F-P and ppc-UTR-lib-R-P primers using pCDF-ppc as a template^9^. The PCR products were blunt-end ligated using T4 DNA ligase (TaKaRa, Kyoto, Japan) at 16°C overnight, and then used to transform the *E. coli* ElectroMAX™ DH5α-E™ strain (Invitrogen, Carlsbad, CA, USA). The purified plasmids were transformed into WLR4 for subsequent enrichment.

The Kan^R^-cassette was amplified from pKAN using gapAHkanF and gapAHkanR primers to enable subsequent deletion of chromosomal *gapA* in HC101 with the Red recombination system using pKD46 and pCP20^18^, as described in a previous study^12^. pCDF-fd-nfor was generated by amplifying *fd* and *nfor* using XbaI-fd-F/XhoI-fd-R and XhoI-nfor-F/BamHI-nfor-R primer pairs, respectively, and inserting the resulting PCR products into the corresponding sites of the modified pCDF vector. pETDuet-gapA was generated by amplifying pETDuet using the 5′-phosphorylated pET-M-F-P and pCDF-pET-M-R-P primers followed by blunt-end ligation. The *gapA* genes were amplified using XbaI-gapA-F and SphI-gapA-R primers and inserted into the *Xba*I and *Sph*I sites of the modified pET vector. To generate 5′-UTR variants, we performed PCR using the resulting construct as a template with 5′-phosphorylated primers (gapA-UTR-lib-F-P and gapA-UTR-lib-R-P). The remaining step was the same as that described above except that the purified, sequence-verified plasmids were transformed into HC102 for hydrogen production.

### Growth and fluorescence measurements

The isolation of *E. coli* DH5α clones containing the various combinations of *sgfp* and *mCherry* variants was manually conducted by using Safe Imager™ 2.0 Blue-Light Transilluminator (Invitrogen, Carlsbad, CA, USA) so that the range of expression levels becomes as broad as possible. They were grown overnight at 37°C in M9 minimal medium containing 4 g/l D-glucose, 0.1% casamino acids, and appropriate antibiotics or inducers using Bioscreen C MBR (Oy Growth Curves Ab, Helsinki, Finland). A fresh 100-well plate containing 200 μl of the same M9 minimal media was then inoculated with triplicate 1:100 dilutions of the overnight cultures. After incubation for 5 h at 37°C with vigorous shaking, 100 μl of each culture was transferred to a 96-well fluorescence-measuring plate, and fluorescence was detected with a VICTOR^3^™ 1420 multilabel counter (PerkinElmer, Wellesley, MA, USA) using a 486-nm excitation filter and a 535-nm emission filter for *sgfp* and a 570-nm excitation filter and a 610-nm emission filter for *mCherry*, both with a 1-s measurement time. The fluorescence intensity depicted in the figures is given using the arbitrary units (a.u.) provided by the instrument, per OD_600_. The non-isolated *E. coli* DH5α library clones were grown as described above and, after washing and resuspending in phosphate-buffered saline (PBS), were analyzed using a two-color fluorescence activated cell sorter (FACSCalibur; BD Biosciences, San Jose, CA, USA) to observe the entire population.

### Lysine and hydrogen production, detection of metabolites, and *gapA* activity assay

The enriched strain for lysine production (WLREU) was grown overnight in complemented M9 medium containing 40 μg/ml of streptomycin and 25 μg/ml of chloramphenicol. Fresh seeds were prepared by diluting overnight cultures to a final OD_600_ of ~0.1 and culturing in the same fresh medium until reaching an OD_600_ of 0.8 (~8 h). The culture broths were inoculated at a final OD_600_ of ~0.1 into 20 ml of complemented M9 media in a 300-ml flask and incubated at 37°C with shaking (200 rpm). The concentration of glucose consumed was determined by high-performance liquid chromatography (HPLC) with an Aminex HPX-87H column (Bio-Rad Laboratories, Richmond, CA, USA) at a flow rate of 0.6 ml/min at 65°C using 5 mM H_2_SO_4_ as the mobile phase. The glucose signal was monitored using a Shodex RI-101 detector (Shodex, Klokkerfaldet, Denmark). The lysine concentration in the broth was determined using a pre-column o-phthalaldehyde (OPA) derivatization method^19^ coupled with a reversed-phase liquid chromatography (LC) column (Acclaim 120 C18; Dionex, Sunnyvale, CA, USA) using an UltiMate 3000 analytical HPLC system (Dionex). Derivatized lysine was eluted at a flow rate of 1.5 ml/min with gradient of acetonitrile:methanol:water solution (v/v % 45:45:10) and 50 mM sodium acetate buffer (pH 6.5), and was detected using a UV-VIS diode array detector at 338 nm. For hydrogen production, cells were grown as described previously^11^ with a modification of supplemented antibiotics, and hydrogen gas evolved after culturing at 25°C for 24 h was analyzed by gas chromatography (Model 6890N, Agilent Technologies, Palo Alto, CA, USA) using a pulsed-discharge ionization detector at 240°C and a Supelco Carboxen-1010 PLOT capillary column (30 m × 0.32 mm) with helium as a carrier gas. To allow measurement of *gapA* activity, cell lysates were prepared using BugBuster from EMD Millipore (Darmstadt, Germany); total protein was measured by Bradford Assay, as described in a previous study^20^. The enzymatic activity of *gapA*-encoded glyceraldehyde-3-phosphate dehydrogenase (GAPDH) was measured using a Colorimetric GAPDH Assay Kit according to the manufacturer's instructions (ScienCell Research Laboratories, CA, USA) and expressed relative to the amount of total protein to yield specific enzymatic activity (Units/mg total protein).

### Statistics and error analysis

Statistical tests (*P*-value) were conducted by Python (SciPy stats). The squared correlation coefficient *R*^2^ for linear regression is calculated by according to *R*^2^ = (NΣ(x_i_y_i_) − Σx_i_Σy_i_)^2^/[(NΣ(x_i_^2^) − (Σx_i_)^2^)(NΣ(y_i_^2^) − (Σy_i_)^2^)], where y = log (expression level) or GAPDH activity, x = ΔG_UTR_ or relative predicted expression level and N = number of data points.

## Author Contributions

S.W.S. and J.-S.Y. designed and performed the experiments, analyzed the data and wrote the manuscript. H.-S.C., J.Y. and S.C.K. performed the experiments. J.P. analyzed the data and edited the manuscript. S.K. and G.Y.J. designed experiments, analyzed the data and wrote the manuscript.

## Supplementary Material

Supplementary Information Supplementary Information
