# Supplementary material for: Predictive combinatorial design of mRNA translation initiation regions for systematic optimization of gene expression levels
Source: Sci Rep. 2014 Mar 31;4:4515. doi: 10.1038/srep04515 (PMC3970122; doi:10.1038/srep04515)
Supplement: Supplementary Information [file srep04515-s1.pdf]

Supplementary Information for:

Predictive combinatorial design of mRNA translation initiation regions for systematic optimization of gene expression levels

Sang Woo Seo[1], Jae-Seong Yang[1], Han-Saem Cho, Jina Yang, Seong Cheol Kim, Jong Moon Park, Sanguk Kim[2], Gyoo Yeol Jung[2]

[1] These authors contributed equally to this work.

[2] To whom correspondence should be addressed.

E-mail: gyjung@postech.ac.kr (G. Y. J.); sukim@postech.ac.kr (S. K.)

Contents:

Supplementary Figures S1-S8

Supplementary Tables S1-S5

Supplementary Methods

Supplementary Figures

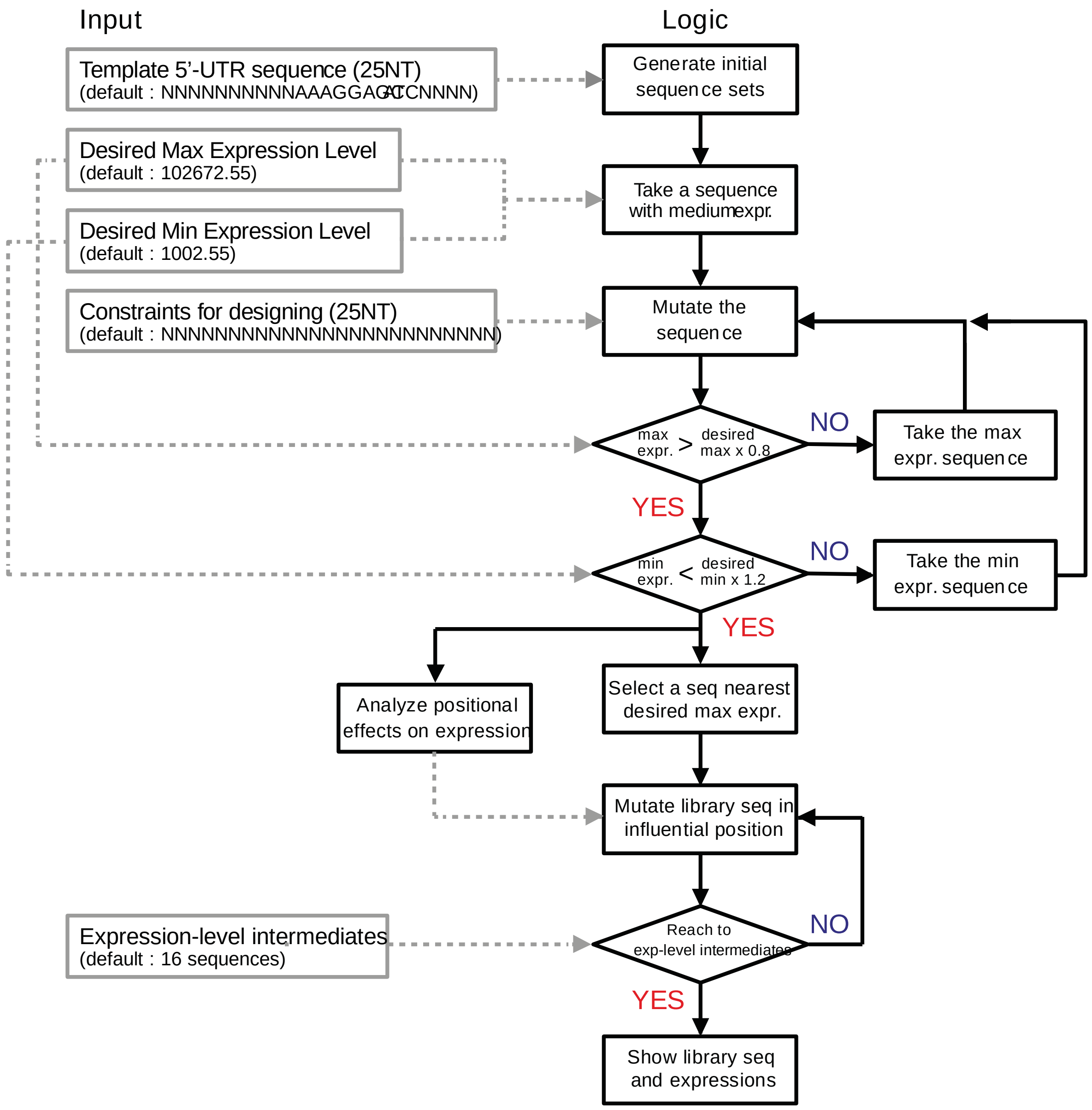

Supplementary Figure S1. Detailed flowchart of UTR Library Designer. The program takes a 5'-UTR template sequence and initially generates randomized sequences. From these, the program selects a medium-expressed sequence. Applying user-defined design constraints, the program then mutates sequences to generate a library that reaches minimum and maximum expression values using a genetic algorithm. If the program finds minimally and maximally expressed sequences, it starts to fit user-defined the number of expression-level intermediates to generate the library. If the number of sequence generated is less than the query, the program gradually adds less influential mutations until it is satisfied. In the opposite situation, the program gradually removes less influential mutations until it is satisfied.

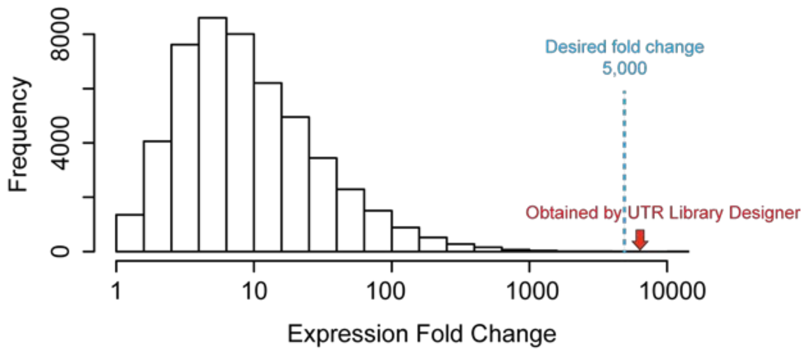

Supplementary Figure S2. Distribution of expression fold-changes in a random library of *gfp* with 16 expression-level intermediates. The utility of UTR Library Designer was compared with that of random trials using *gfp* as a target. We attempted to obtain a library containing 50,000 different sets of sequences that satisfied minimum and maximum expressions of 40 and 200,000, respectively, with 16 expression-level intermediates. Our desired expression fold-change was 5,000 (200,000/40), a goal effectively out of reach of the random approach. The red arrow indicates the average value of 10 trials using UTR Library Designer.

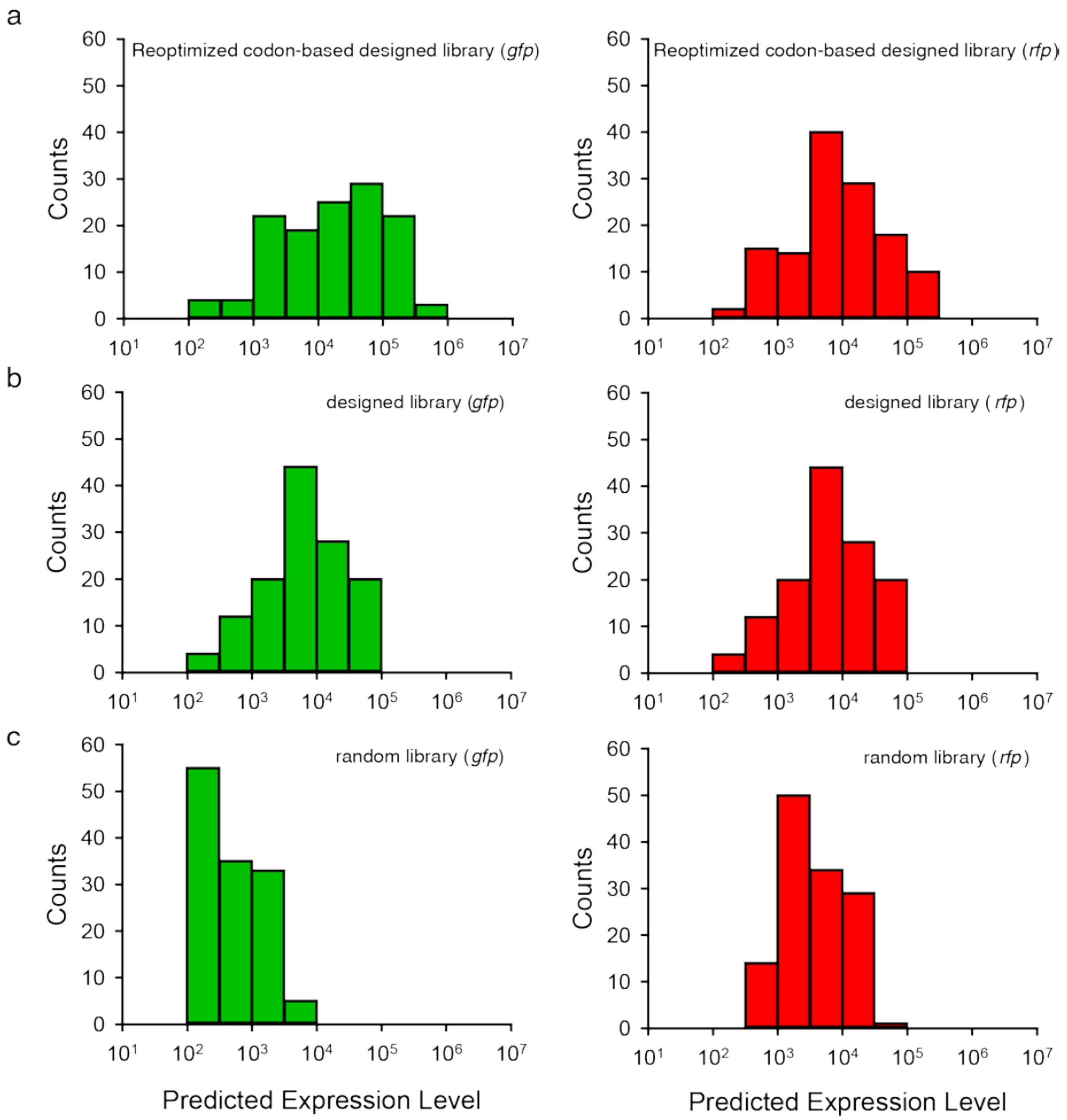

Supplementary Figure S3. *In silico* prediction of the expression level of the designed 5'-UTR libraries. The range of predicted expression levels of 5'-UTR libraries (128 each) for each reporter gene differed depending on the methods. (a) Reoptimized codon-based designed library; (b) designed library; (c) random library.

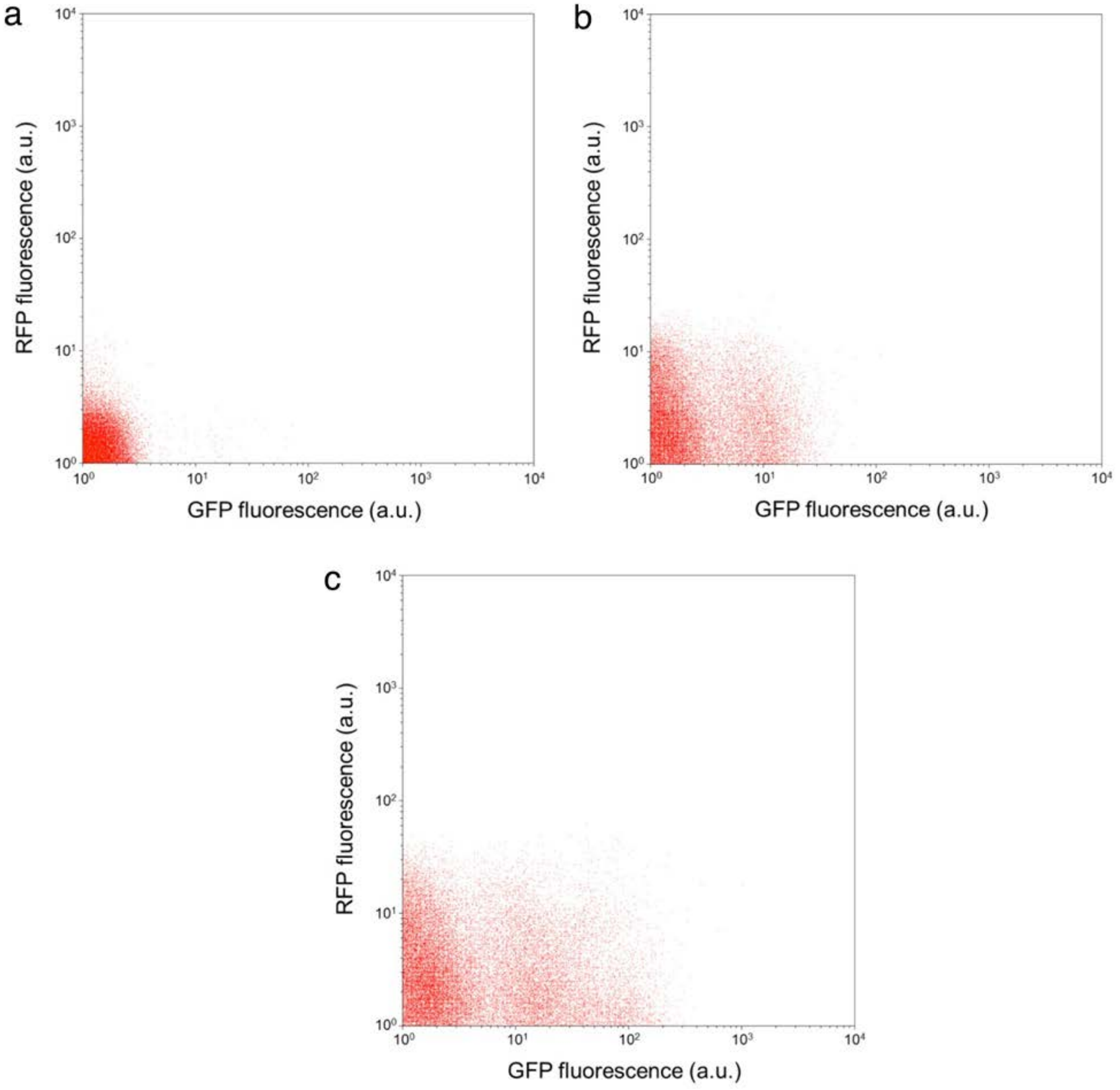

Supplementary Figure S4. *In vivo* validation of the designed 5'-UTR libraries by FACS. The range of the expression levels of designed variants was similar to the *in silico*-predicted range of expression level for each design method. (a) random library; (b) designed library; (c) reoptimized codon-based designed library.

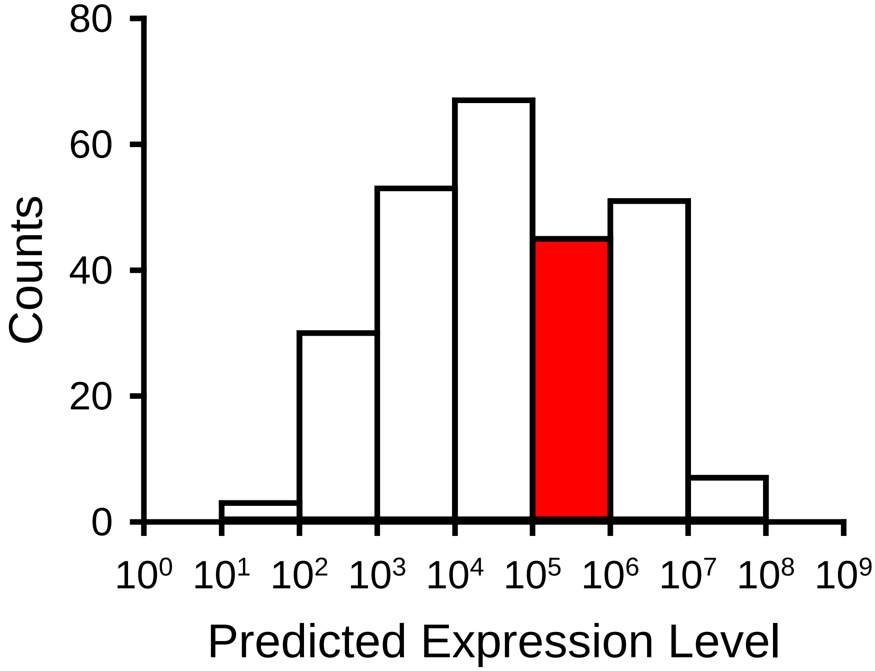

Supplementary Figure S5. The distribution of predicted expression levels of the designed 5'-UTR library for the expression of *ppc*. The designed 5'-UTR library for *ppc* (256 variants) was predicted to show more than a 10^5-fold range in expression level. The enriched variant for lysine production belongs to the bar, which was hardly able to be found using a random search.

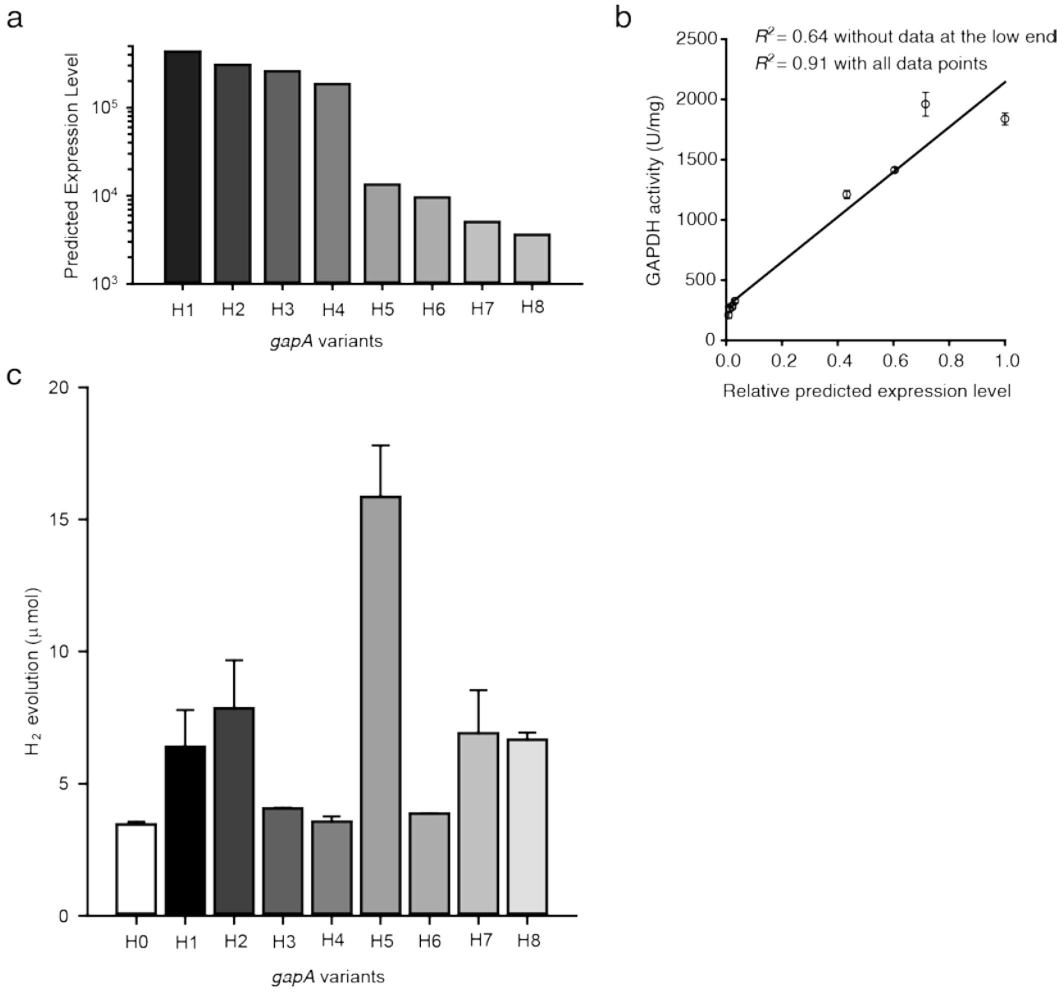

Supplementary Figure S6. Pathway optimization for hydrogen production by changing *gapA* expression. (**a**) The predicted expression level of the designed 5’-UTR library for *gapA* expression. (**b**) The linear correlation between the predicted expression level and specific enzymatic activity. (**c**) The amount of hydrogen evolved by each variant. H0 control stands for the wild-type *gapA* expression from chromosome of W3110 with native regulatory system but has empty vectors.

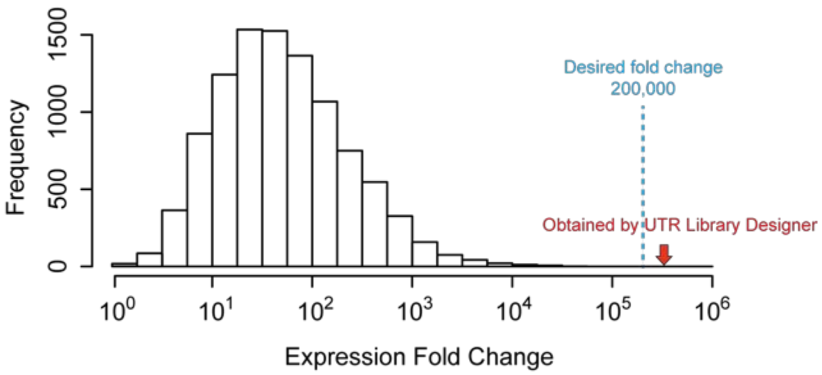

Supplementary Figure S7. Distribution of expression fold-changes in a random library of *ppc* with 256 expression-level intermediates. The utility of UTR Library Designer was compared with that of random trials using *ppc* as a target. We attempted to obtain a library containing 10,000 different sets of sequences that satisfied minimum and maximum expressions of 50 and 10,000,000, respectively, with 256 expression-level intermediates. Our desired expression fold-change was 200,000 (10,000,000/50), a goal effectively out of reach of the random approach. The red arrow indicates the value obtained by UTR Library Designer for *ppc* optimization.

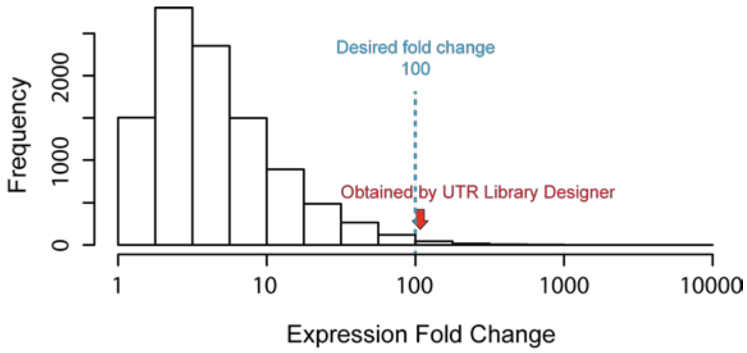

Supplementary Figure S8. Distribution of expression fold changes in a random library of *gapA* with 8 expression-level intermediates. The utility of UTR Library Designer was compared with that of random trials using *gapA* as a target. We attempted to obtain a library containing 10,000 different sets of sequences that satisfied minimum and maximum expressions of 5,000 and 500,000, respectively, with 8 expression-level intermediates. Our desired expression fold-change was 100 (500,000/5,000), a goal effectively out of reach of the random approach. The red arrow indicates the value obtained by UTR Library Designer for *gapA* optimization.

Supplementary Tables

Supplementary Table S1. Statistics for the output of UTR Library Designer to obtain a 5,000-fold change in expression level with 16 expression-level intermediates. Red colors indicate variants tested *in vivo* as shown in Fig. 2.

Input						Output					
Template 5'-UTR Sequence	Additional Constraints	Protein Coding Sequence (N-term 35NT)	Min. Expr.	Max. Expr.	Expression-level intermediates	Library UTR Sequence	# of trial	Min. Expr.	Max. Expr.	Expression-level intermediates	Output URL
NNNNNNNNNNAAAGGAGCATCNNNN	NNNNNNNNNNNNNNNNNNNNNNNNN	ATGGCTAGCAAGGGCGAGGAGCTGTTCACCGGGGT	40	200,000	16	CCTRTTGTCTAAAGKAGSATCGCCM	119	36.83	197,107.41	16	http://sbi.postech.ac.kr/utr_library/job/1368054392-370/
NNNNNNNNNNAAAGGAGCATCNNNN	NNNNNNNNNNNNNNNNNNNNNNNNN	ATGGCTAGCAAGGGCGAGGAGCTGTTCACCGGGGT	40	200,000	16	CGATTGCCCYWAAGSAGSATCGCGG	96	48.71	186,385.82	16	http://sbi.postech.ac.kr/utr_library/job/1368075996-140/
NNNNNNNNNNAAAGGAGCATCNNNN	NNNNNNNNNNNNNNNNNNNNNNNNN	ATGGCTAGCAAGGGCGAGGAGCTGTTCACCGGGGT	40	200,000	16	CGAACCGTCSAAAGSAGSAKCTCCA	150	46.06	268,101.01	16	http://sbi.postech.ac.kr/utr_library/job/1368075972-090/
NNNNNNNNNNAAAGGAGCATCNNNN	NNNNNNNNNNNNNNNNNNNNNNNNN	ATGGCTAGCAAGGGCGAGGAGCTGTTCACCGGGGT	40	200,000	16	GTCCATGTGCAAAKSAGSATCCGMG	169	48.71	186,385.82	16	http://sbi.postech.ac.kr/utr_library/job/1368138090-80/
NNNNNNNNNNAAAGGAGCATCNNNN	NNNNNNNNNNNNNNNNNNNNNNNNN	ATGGCTAGCAAGGGCGAGGAGCTGTTCACCGGGGT	40	200,000	16	GCTGMCAGAGAAAGSAGCRTCMTTG	134	38.95	226,687.82	16	http://sbi.postech.ac.kr/utr_library/job/13680759

											19-820/
NNNNNNNNNNAAAGGAGCATCNNNN	NNNNNNNNNNNNNNNNNNNNNNNNN	ATGGCTAGCAAGGGCGAGGAGCTGTTCACCGGGGT	40	200,000	16	TTTCTTCGCCAAAGSAGSWWCGCGC	142	34.82	375,006.67	16	http://sbi.postech.ac.kr/utr_library/job/1368139207-580/
NNNNNNNNNNAAAGGAGCATCNNNN	NNNNNNNNNNNNNNNNNNNNNNNNN	ATGGCTAGCAAGGGCGAGGAGCTGTTCACCGGGGT	40	200,000	16	CKTTTCCCTTAWAGSAGSATCCGGG	99	41.19	166,660.5	16	http://sbi.postech.ac.kr/utr_library/job/1368139202-450/
NNNNNNNNNNAAAGGAGCATCNNNN	NNNNNNNNNNNNNNNNNNNNNNNNN	ATGGCTAGCAAGGGCGAGGAGCTGTTCACCGGGGT	40	200,000	16	TACGGGCTCAAAWGSAGCMTCMCAC	163	38.95	95,264.65	16	http://sbi.postech.ac.kr/utr_library/job/1368139193-570/
NNNNNNNNNNAAAGGAGCATCNNNN	NNNNNNNNNNNNNNNNNNNNNNNNN	ATGGCTAGCAAGGGCGAGGAGCTGTTCACCGGGGT	40	200,000	16	CGWCGCGCCCAAAGKAGSATCMCCG	173	41.19	149,022.73	16	http://sbi.postech.ac.kr/utr_library/job/1368138910-210/
NNNNNNNNNNAAAGGAGCATCNNNN	NNNNNNNNNNNNNNNNNNNNNNNNN	ATGGCTAGCAAGGGCGAGGAGCTGTTCACCGGGGT	40	200,000	16	GCTTGGTCGCAAAGSAGSAMCGRGA	102	32.93	570,446.18	16	http://sbi.postech.ac.kr/utr_library/job/1368139113-830/

Supplementary Table S2. Statistics for random mutations to obtain a 5,000-fold change in expression level with 16 expression-level intermediates

# of Trials for library to have >5,000-fold changes	Min Expression Obtained	Max Expression Obtained	Fold Change	Expression-level intermediates
12912	18.37	104182.78	5672.71	16
Not found (within 80,000 trials)	-	-	-	16
43936	12.73	166660.50	13096.95	16
Not found (within 80,000 trials)	-	-	-	16
Not found (within 80,000 trials)	-	-	-	16
14512	3.40	19219.82	5660.03	16
Not found (within 80,000 trials)	-	-	-	16
Not found (within 80,000 trials)	-	-	-	16
23696	3.30	16730.54	5061.02	16
Not found (within 80,000 trials)	-	-	-	16

Supplementary Table S3. Statistics for random mutations to obtain a 2,000-fold change in expression level with 16 expression-level intermediates

# of Trials for library to have >2,000-fold changes	Min Expression Obtained	Max Expression Obtained	Fold Change	Expression-level intermediates
12928	18.37	104182.78	5672.71	16
75200	76.45	318858.31	4170.57	16
43952	12.73	166660.50	13096.95	16
38496	7.41	18502.57	2497.66	16
41728	20.02	72024.68	3598.07	16
14528	3.40	19219.82	5660.03	16
54896	86.72	237415.88	2737.64	16
23360	0.59	2051.85	3494.93	16
19312	11.81	26064.61	2207.69	16
5328	8.23	29976.19	3643.99	16

Supplementary Table S4. Strains and plasmids used in this study.

Name	Relevant characteristics	Source
**Strains**		
Mach1-T1^R	F^- φ80(*lac*Z)ΔM15 Δ*lac*X74 *hsd*R(r_K^- m_K^+) Δ*rec*A1398 *end*A1 *ton*A	Invitrogen
DH5α	F- ϕ80*lac*ZΔM15 Δ(*lac*ZYA-*arg*F) U169 *rec*A1 *end*A1 *hsd*R17 (r_k^-, m_k^+) *gal*$^-$ *pho*A *sup*E44 λ^-*thi*$^-$1 *gyr*A96 *rel*A1	Invitrogen
HC101	BL21(DE3) Δ*ldh*A::FRT Δ*sth*A::FRT Δ*hya*B::*hyd*AE-FRT Δ*hyb*C::*hyd*FG-FRT	Ref. 12
HC102	HC101 Δ*gap*A::FRT	This study
H0	HC101 with pCDF-Fd-NFOR, pACYCDuet, pETDuet	This study
H1	HC102 with pCDF-Fd-NFOR, pA2221, pETDuet-gapA1	This study
H2	HC102 with pCDF-Fd-NFOR, pA2221, pETDuet-gapA2	This study
H3	HC102 with pCDF-Fd-NFOR, pA2221, pETDuet-gapA3	This study
H4	HC102 with pCDF-Fd-NFOR, pA2221, pETDuet-gapA4	This study
H5	HC102 with pCDF-Fd-NFOR, pA2221, pETDuet-gapA5	This study
H6	HC102 with pCDF-Fd-NFOR, pA2221, pETDuet-gapA6	This study
H7	HC102 with pCDF-Fd-Nfor, pA2221, pETDuet-gapA7	This study
H8	HC102 with pCDF-Fd-NFOR, pA2221, pETDuet-gapA8	This study
W3110	F^- λ^- *rph-1 IN*(*rrnD, rrnE*)*1*	ATCC 27325
WL3	W3110 *lys*C::BBa_J23100_*lys*C^{fbr} P_{dapA}::BBa_J23100 P_{dapB}::BBa_J23100 P_{lysA}::BBa_J23100 *lac*ZYA::BBa_J23100-*ddh* Δ*met*L Δ*thr*A Δ*icl*R Δ*icl*R	Ref. 9
WLR4	WL3 Δ*ppc* with LysRibo	Ref. 9
WLREU	WLR4 with pCDF-WLREUppc	This study
**Plasmids**		
pKD46	Red recombinase expression vector; Amp^R	Ref. 18
pCP20	FLP expression vector; Amp^R	Ref. 18
pACYCDuet	Expression vector, Cm^R, p15A ori	Novagen
pCDFDuet	Expression vector, Sm^R, cloDF13 ori	Novagen
pETDuet	Expression vector, Amp^R, ColE1 ori	Novagen

pKAN	pET101/D-TOPO with Kan^R-cassette	Ref. 12
pACYC-sgfp	R14	Ref. 5
pACYC-sgfpOpt	R20	Ref. 5
pCDF-mCherry	pCDFm-*Xba*I-*mCherry*-*Sph*I	This study
pCDF-mCherryOpt	pCDFm-*Xba*I-*mCherryOpt*-*Sph*I	This study
pCDF-fd-nfor	pCDFm-*Xba*I-*fd*-*Xho*I-*nfor*-*BamH*I	This study
pETDuet-gapA	pETm-*Xba*I-*gapA*-*Sph*I	This study
pA2221	pACYDDuet-1 with mutant2221 GAPDH	Ref. 12
pETDuet-gapA1	pETm-*Xba*I-GTTTACACTCAAAGGAGCATATTAC-*gapA*-*Sph*I	This study
pETDuet-gapA2	pETm-*Xba*I-BBa_J23100-GTTTACACTCAAAGGAGCATCTTAC-*gapA*-*Sph*I	This study
pETDuet-gapA3	pETm-*Xba*I-BBa_J23100-GTTTCCACTCAAAGGAGCATATTAC-*gapA*-SphI	This study
pETDuet-gapA4	pETm-*Xba*I-BBa_J23100-GTTTCCACTCAAAGGAGCATCTTAC-*gapA*-SphI	This study
pETDuet-gapA5	pETm-*Xba*I-BBa_J23100-GTTTACACTCAAAGAAGCATATTAC-*gapA*-SphI	This study
pETDuet-gapA6	pETm-*Xba*I-BBa_J23100-GTTTACACTCAAAGAAGCATCTTAC-*gapA*-SphI	This study
pETDuet-gapA7	pETm-*Xba*I-BBa_J23100-GTTTCCACTCAAAGAAGCATATTAC-*gapA*-SphI	This study
pETDuet-gapA8	pETm-*Xba*I-BBa_J23100-GTTTCCACTCAAAGAAGCATCTTAC-*gapA*-SphI	This study
LysRibo	pACYCDuet-*KpnI*-BBa_J23100-*lysC UTR*-*tetA*-*SacI*	Ref. 9
pCDF-ppc	pCDFDuet-*KpnI*-*ppc*-*SacI*	Ref. 9
pCDF-WLREUppc	pCDFDuet-*KpnI*-BBa_J23100-TATCTGCGAAAACTCGGAGCTACAC-*ppc*-*SacI*	This study

[a] Red letters indicate the region of variations in 5'-UTR sequences.

Supplementary Table S5. Primers used in this study.

Name	Sequence (5'-3')[a,b,c]
pCDF-M-F-P	aaaaaaaaaccccgcccctgacagggcggggtttttttaccctgccctgaaccgac
pCDF-pET-M-R-P	acgatGCATGCgtacgattCCATGGtaagcctaGAATTCgttagctaCTCGAGaatctcaaTCTAGAcctaatgcaggagtcgcataaggg
pCDF-del-XbaI-F	tactgaaccgctcttgatttcagtgcaat
pCDF-del-XbaI-R	attgcactgaaatcaagagcggttcagta
XbaI-mCherry-F	aTCTAGAatggtctccaagggcgaggaggacaatatggctatcattaaagagttcatgcgc
SphI-mCherry-R	aGCATGCcttaactgttatgtcgactcagagg
XbaI-mCherryOpt-F	aTCTAGAatggtttccaagggcgagg
sgfpOpt-16bp-1-F-P	ttgacggctagctcagtcctaggtacagtgctagcCCTRTTGTCTAAAGKAGSATCGCCMatggctagcaagggcgaggag
sgfpOpt-16bp-5-F-P	ttgacggctagctcagtcctaggtacagtgctagcGCTGMCAGAGAAAGSAGCRTCMTTGatggctagcaagggcgaggag
sgfp-Random-F-P	ttgacggctagctcagtcctaggtacagtgctagcCTGCGACTATAACGCAGNNNNNGGAatggccagcaagggcgagg
sgfp-Designed-F-P	ttgacggctagctcagtcctaggtacagtgctagcGTTTMCAMTCWAAGRAGCAKCKTMCatggccagcaagggcgagg
sgfpOpt-ReoptCodonDesigned-F-P	ttgacggctagctcagtcctaggtacagtgctagcGTTTMCAMTCAWAGGMGYAYCTTRCatggctagcaagggcgaggag
mCheery-Random-F-P	ttgacggctagctcagtcctaggtacagtgctagcGTTTCCGCCCAATGGAGNNNNNTACatggtctccaagggcgaggag
mCheery-Designed-F-P	ttgacggctagctcagtcctaggtacagtgctagcGTTTCCRMYCAAWGGAGCSWYTTACatggtctccaagggcgaggag
mCherryOpt-ReoptCodonDesigned-F-P	ttgacggctagctcagtcctaggtacagtgctagcGTTTYCRCTCAAAGRAGCMTMTTMMatggtttccaagggcgagg
sGFP-mCherry-lib-R-P	gcgcaacgcaattaatgtaagttagc
Del-gapA-F	gtaattttacaggcaaccttttattcactaacaaatagctggtggaatattaattttgtttaactttaagaagga
Del-gapA-R	ctctttttagatcacagtgtcatctcaacttatttggagatgtgagcgatctcaatggtgatggtgatgatgacc
XbaI-fd-F	aTCTAGAggaattgtgagcggataacaattgacattgtgagcggataacaagatactgagcacaggatcccacaaaggagcatctactatggcatataaaatcgctgattcatg
XhoI-fd-R	cctttcacCTCGAGtttattcttgtactggtgctccaac
XhoI-nfor-F	aCTCGAGgtgaaaggagcaaaataaatgagggaagacacaaaggtgtacgacataacgattataggcgggggaccggt
BamHI-nfor-R	accgtgtgcttctcaaatgcctgagaaaaaaaaccccgccctgtcaggggcggggtttttttttGAATTCcagcgatcgcgtggccg
pET-M-F-P	gctgaaaggaggaactatatccgg
XbaI-gapA-F	aTCTAGAatgactatcaaagtaggtatcaacgg

SphI-gapA-R	aGCATGCaaaatgccgccagccgaactgg
gapA-UTR-lib-F-P	ttgacggctagctcagtcctaggtacagtgctagcGTTTMCACTCAAAGRAGCATMTTACatgactatcaaagtaggtatcaacgg
gapA-UTR-lib-R-P	tctagacctaatgcaggagtc
ppc-UTR-lib-F-P	ttgacagctagctcagtcctagggattgtgctagcTAKCTGCGAAAACWMGSAGSWAMRCatgaacgaacaatattccgcattgcgtag
ppc-UTR-lib-R-P	ccgagctcggtaccctcgagtctggtaaag

[a] Capital letters indicate restriction sites.

[b] Underlined letters indicate homologous sequences for recombination.

[c] Underlined and capital letters indicate 5’-UTR sequences for library construction.

Supplementary Materials and Methods

Comparison of UTR Library Designer with random trials

We tested how fast UTR Library Designer could reach a desired gene expression range compared to a random search using *gfp* as a target. We ran UTR Library Designer 10 times under 16 expression-level intermediates conditions, setting 40 and 200,000 as minimum and maximum expression levels, respectively. In random searches, we changed 5'-UTR sequences to allow mutations to create 16 sequences. Ten different test sets with 5,000 trials in each set were conducted in parallel (50,000 trials for random searches). Statistics for UTR Library Designer and random trials are depicted in Supplementary Figure S2 and summarized in Supplementary Tables S1, S2, and S3. We also tested random trials using other genes (*ppc* and *gapA*) that were targets for pathway optimization, applying the same constraints as used for UTR Library Designer (Supplementary Figure S7 and S8).
